# Association between Non-Steroidal Anti-Inflammatory Drug Use and Major Cardiovascular Outcomes in Patients with Acute Coronary Syndrome in the Arabian Gulf

**DOI:** 10.3390/jcm12175446

**Published:** 2023-08-22

**Authors:** Ibrahim Al-Zakwani, Juhaina Salim Al-Maqbali, Wael AlMahmeed, Najib AlRawahi, Abdullah Al-Asmi, Mohammad Zubaid

**Affiliations:** 1 Department of Pharmacology & Clinical Pharmacy, College of Medicine & Health Sciences, Sultan Qaboos University, Muscat 123, Oman; juhaina@squ.edu.om; 2 Department of Pharmacy, Sultan Qaboos University Hospital, Muscat 123, Oman; 3 Gulf Health Research, Muscat 111, Oman; 4 Heart & Vascular Institute, Cleveland Clinic, Abu Dhabi 112412, United Arab Emirates; wmahmeed@gmail.com; 5 National Heart Center, Royal Hospital, Muscat 111, Oman; nalrawahi@yahoo.com; 6 Department of Medicine, College of Medicine & Health Sciences, Sultan Qaboos University, Muscat 123, Oman; alasm@squ.edu.om; 7 Department of Medicine, Faculty of Medicine, Kuwait University, Safat 24923, Kuwait; mohammed.zubaid@ku.edu.kw

**Keywords:** non-steroidal anti-inflammatory drug, stroke, acute coronary syndrome, myocardial infarction, mortality, readmission

## Abstract

*Objectives*: Studies on the association between non-steroidal anti-inflammatory drugs (NSAIDs) and major adverse cardiovascular events (MACE) in the Arabian Gulf are scarce. The aim of this study was to evaluate the association between NSAIDs use and MACE in acute coronary syndrome (ACS) patients in the Arabian Gulf region. *Methods:* Data were analyzed from 3007 consecutive patients diagnosed with ACS admitted to 29 hospitals in four Arabian Gulf countries from January 2012 to January 2013, as well as being on prior NSAIDs use during the *index* admission. The MACE included stroke/transient ischemic attacks (TIAs), myocardial infarction (MI), all-cause mortality and readmissions for cardiac reasons. *Results*: The overall mean age of the cohort was 62 ± 12 years, and 9.6% (*n* = 290) of the patients were on *prior* NSAID use during the *index* admission. At 12-months follow-up, after adjusting for confounding factors, patients on NSAIDs were significantly more likely to have had MACE (adjusted OR (aOR), 1.89; 95% confidence interval (CI): 1.44–2.48; *p* < 0.001). Specifically, the higher event rates observed were stroke/TIA (aOR, 2.50; 95% CI: 1.51–4.14; *p* < 0.001) and readmissions for cardiac reasons (aOR, 2.09; 95% CI: 1.59–2.74; *p* < 0.001), but not MI (aOR, 1.26; 95% CI: 0.80–1.99; *p* = 0.320) and all-cause mortality (aOR, 0.79; 95% CI: 0.46–1.34; *p* = 0.383). *Conclusions*: NSAIDs use was associated with significant stroke/TIA events as well as readmissions for cardiac reasons. However, NSAIDs were not associated with increased MIs or all-cause mortality rates in patients with ACS in the Arabian Gulf.

## 1. Introduction

Acute coronary syndrome (ACS) is one of the leading causes of morbidity and mortality in Asia as well as globally [1,2], accounting for approximately seven million deaths and 129 million disability-adjusted life years (DALYs) annually worldwide [3,4]. ACS is also associated with significant economic burden on both direct and indirect healthcare costs, with economic analyses suggesting that hospitalizations and readmissions for ACS account for 60–90% of total annual health care costs [5,6,7].

Non-steroidal anti-inflammatory drugs (NSAIDs) are one of a group of medications that is widely used and easily available over the counter (OTC). More than 70 million prescriptions for NSAIDs are written annually, and taking into account OTC medications, 30 billion doses of NSAIDs are consumed annually in the United States alone [8]. They are used, in the short- and long-term, for a variety of indications, including pain and rheumatoid arthritis, amongst many others. Besides gastrointestinal bleeding, which is partially alleviated by the use of proton pump inhibitors (PPIs), NSAIDs have also been associated with major adverse cardiovascular events (MACE) including stroke [9,10]. The cardiovascular risk may not only be limited to non-selective NSAIDS but also to selective cyclooxygenase 2 (COX-2) inhibitors [11].

The fact that NSAIDs are widely accessible worldwide coupled with their undesirable association with gastrointestinal, renal and cardiovascular side effects makes its continued investigation warranted. There is currently limited research on the use of NSAIDs in ACS population not only in the Arabian Gulf, but in the world at large. Hence, the aim of this study was to evaluate the association between NSAIDs use and MACE, including readmissions for cardiac reasons in patients with ACS in the Arabian Gulf.

## 2. Methods

The Gulf COAST registry methods have already been previously reported [12]. In summary, the Gulf COAST registry was a prospective, multicenter, multinational, longitudinal cohort study of consecutive citizens from the Arabian Gulf (Bahrain, Kuwait, Oman and United Arab Emirates) admitted to 29 hospitals with a diagnosis of ACS from January 2012 to January 2013. The registry enrolled a total of 4044 patients who were ≥18 years of age with ACS diagnosed according to American College of Cardiology (ACC) clinical data standards [13]. All other diagnoses, including stroke, transient ischemic attack, myocardial infarction, mortality, diabetes mellitus, hypertension, dyslipidemia and heart failure, were also based on key data elements as defined by the ACC clinical data standards [13]. Education was defined as any formal education from primary school and above. Alcohol use was defined as low as any drink/s per week. Apart from excluding non-citizens as well as patients who were not willing/able to provide consent, there were no other exclusion criteria. The study was approved by the local institutional ethics committees of participating centers in the four Arabian Gulf countries.

Data collected included patient demographics (age, gender, employment status, marital status, education status, health insurance, body mass index (BMI), tobacco and alcohol use), medical history and risk factors related to MACE, prior medication use, laboratory data, clinical presentation and management during hospital stay including medications, reperfusion therapy and procedures and discharge medications. Follow up was performed at 12-months from the date of enrolment, and was carried out via clinic visits or telephone interviews.

The main predictor variable was NSAID use, while the outcomes collected included 12-months cumulative stroke/transient ischemic attack (TIA), myocardial infarction (MI), all-cause mortality and readmissions for cardiac reasons as well as overall MACE.

### Statistical Analysis

For categorical variables, frequencies and percentages were reported. Differences among groups were analyzed using Pearson’s χ^2^ tests (or Fisher’s exact tests for expected cells of <5). For continuous variables, mean and standard deviation were used to present the data while analyses were performed using Student’s *t*-test. Continuous variables that were not normally distributed were summarized as median and interquartile range, and analyses were conducted using Wilcoxon–Mann–Whitney tests. The association between NSAID use and MACE (stroke/TIA, MI, all-cause mortality, readmissions for cardiac reasons and overall MACE) was evaluated via multivariate logistic regression utilizing the simultaneous method and adjusting for the GRACE risk score for in-hospital mortality, which has been validated in an Arabian Gulf ACS Registry [14]. Apart from the GRACE risk score variables (which are derived from age, heart rate, systolic blood pressure (BP), serum creatinine, cardiac arrest at admission, ST segment deviation on EKG, abnormal cardiac enzymes and Killip class), the logistic models were also adjusted for gender, smoking status, marital status, employment status, education status, BMI, diabetes mellitus, peripheral artery disease, percutaneous coronary intervention (PCI)/coronary artery bypass graft (CABG), prior event and use of evidence-based cardiac medications at hospital discharge (aspirin, clopidogrel, beta blocker, statin, angiotensin converting enzyme inhibitor (ACEI) or angiotensin receptor blocker (ARB)).

The goodness-of-fit of the multivariable logistic models was examined using the Hosmer–Lemeshow goodness-of-fit statistic [15] as well as the *C*-index [16]. An a priori two-tailed level of significance was set at the 0.05 level. Statistical analyses were conducted using STATA version 16.1 (StataCorp., 2013, Stata Statistical Software, College Station, TX, USA).

## 3. Results

Out of the 4044 subjects enrolled by the Gulf COAST registry, the present analysis only included patients with non-missing NSAID information (N = 3007). A total of 9.6% (n = 290) of the patients were on prior NSAID use during the index admission. The overall mean age of the cohort was 62 ± 12 years, of which 62% (n = 1851) were males. A total of 22% (n = 647) of the patients were employed and 83% (n = 2495) were married. Thirty-four percent of the patients (n = 1037) were current or prior smokers, and 2.9% (n = 87) were alcohol consumers. Comorbid conditions were common, particularly hypertension (81%; n = 2433), dyslipidemia (70%; n = 2101) and diabetes mellitus (63%; n = 1903).

As shown in Table 1, patients on NSAIDs (compared to patients not on NSAIDs) were more likely to be female (44% vs. 38%; *p* = 0.049), educated (54% vs. 46%; *p* = 0.013), associated with dyslipidemia (77% vs. 69%; *p* = 0.009), hypertension (86% vs. 80%; *p* = 0.016) and heart failure (20% vs. 14%; *p* = 0.012). However, patients on NSAIDs were less likely to be associated with diabetes mellitus (57% vs. 64%; *p* = 0.018) and non-ST-elevation MI (41% vs. 52%; *p <* 0.001). 

Table 2 shows medication utilization prior to admission and the post-discharge data are stratified by NSAIDs use. Prior to the *index* admission, patients on NSAIDs were also more likely to have been on aspirin (85% vs. 79%; *p* = 0.023) and clopidogrel (35% vs. 28%; *p* = 0.015). While 98% (n = 2705) of the cohort was treated optimally with the dual antiplatelet combination (aspirin and clopidogrel concurrently), only 52% (n = 1427) of the patients were prescribed the 5-drug regimen (aspirin, clopidogrel, ACEI/ARB, statin, beta blocker) concurrently, which was significantly higher among patients on NSAIDs than those not on NSAIDs (62% vs. 51%; *p* = 0.001).

The overall MACE rate was 41.1% (n = 1195), with significant differences among the groups as shown in Table 3. Adjusting for demographic and clinical characteristics as well as socioeconomic measures (insurance type, employment, education and marital status), at 12-months follow-up, patients on NSAIDs were significantly more likely to have had MACE (adjusted OR (aOR), 1.89; 95% confidence interval (CI): 1.44–2.48; *p* < 0.001). The higher event rates were specifically observed in stroke/TIA (aOR, 2.50; 95% CI: 1.51–4.14; *p* < 0.001) and in readmissions for cardiac reasons (aOR, 2.09; 95% CI: 1.59–2.74; *p* < 0.001), but not in MI (aOR, 1.26; 95% CI: 0.80–1.99; *p* = 0.320) and 12-months all-cause mortality (aOR, 0.79; 95% CI: 0.46–1.34; *p* = 0.383).

## 4. Discussion

To the best of our knowledge, this is the only study in the Arabian Gulf to have evaluated the association between NSAIDs use and the development of MACE in patients with ACS. At 12-months follow-up, patients on prior use of NSAIDs were associated with increased risk of stroke/TIA events and readmissions for cardiac reasons when compared to patients who were not on NSAIDs. However, NSAIDs use was not associated with increased MI or all-cause mortality rates in patients with ACS in the Arabian Gulf.

Similar to the current findings, a number of other meta-analyses and review articles have also reported that the use of various types of NSAIDs (both non-selective as well as COX-2 inhibitors) are associated with the development of stroke/TIA events compared to patients not using NSAIDs [17,18,19,20,21,22]. The potential mechanisms for NSAID-associated increase in stroke risk are hypothesized to be due to vasoconstriction secondary to inhibition of prostacyclin-induced vasodilation, hypertension induced by direct renal effects on sodium excretion leading to volume expansion and thrombosis due to prostaglandin-mediated platelet aggregation [23]. This study did not document the actual types of NSAIDs used, and this shortcoming may pose a significant limitation, as some have been more associated with stroke/TIA events than others [18]. Furthermore, this study also did not report the duration of use of NSAIDs. However, no safe window on concomitant use of NSAIDs has been reported, with even short-term (0–3 days) use being associated with increased risk of bleeding compared with no NSAIDs use [24].

Our findings showed a significant association between the use of NSAIDs in patients with ACS and readmissions due to cardiac reasons, which can be partly explained by the type, dose and duration of use of NSAIDs, which were not documented in our study. Furthermore, the risk of cardiac complications is higher in the first week of NSAIDs use, but not for all types of NSAIDs, as reported in some studies [10]. A population-based matched case–control study in Finland [25] showed that hospitalizations due to myocardial infarctions in patients using NSAIDs accounted for 17,000 hospitalizations annually. The current study did not show any differences in myocardial infarction rates between the groups, not only at baseline (Table 1), but also at 1-year follow-up (Table 3); however, the NSAIDs group was associated with a higher prevalence of hypertension and heart failure (Table 1), which has been reported as the main reason behind readmissions in patients with ACS [26].

In conclusion, NSAIDs use was associated with a significant increase in stroke/TIA events, as well as readmissions for cardiac reasons. However, NSAIDs were not associated with increased MI or all-cause mortality rates in patients with ACS in the Arabian Gulf. These findings should be interpreted in light of the study’s limitations.

## 5. Limitations

As this was a retrospective study and the fact that the analyses were also adjusted for various confounding factors, bias could still have existed between the groups, as we were not able to control for unmeasured confounding variables. Instead of all-cause mortality, it would have been more pertinent to have reported cardiovascular mortality. Even though all types of NSAIDs are implicated in cardiovascular events, it would have been more informative to have reported the different types of NSAIDs involved. Furthermore, NSAIDs use was only reported during the index hospital admission; they could have been stopped or changed during the 1-year follow-up. However, as reported earlier, there is no safe window on the concomitant use of NSAIDs; even a short-term use period of a few days has been associated with an increased risk of bleeding [24]. A total of 3.2% (n = 97) of the subjects were lost to follow-up at 12-months, and this could have biased the outcomes; however, there were no significant differences in the demographic and clinical characteristics between the patients that were lost to follow-up against the group that remained during the 12-months follow-up period (Table 4).

## Figures and Tables

**Table 1:** Demographic and clinical characteristics of the acute coronary syndrome cohort stratified by non-steroidal anti-inflammatory drug (NSAID) use.

Characteristic, *n (%) Unless Specified Otherwise*		All (N = 3007)	NSAID Use		*p*-Value
			No (n = 2717)	Yes (n = 290)	
*Demographic*					
Age, mean ± SD, years		62 ± 12	62 ± 12	63 ± 12	0.124
Female gender		1156 (38%)	1029 (38%)	127 (44%)	0.049
Educated		1410 (47%)	1254 (46%)	156 (54%)	0.013
Employed		647 (22%)	592 (22%)	55 (19%)	0.266
Married		2495 (83%)	2252 (83%)	243 (84%)	0.696
BMI, mean ± SD, kg/m^2^		29.1 ± 7.0	29.1 ± 7.1	28.7 ± 6.3	0.305
Smoking (current or prior)		1037 (34%)	939 (35%)	98 (34%)	0.794
Alcohol		87 (2.9%)	75 (2.8%)	12 (4.1%)	0.183
*Past medical history*					
Prior MI		1026 (34%)	933 (34%)	93 (32%)	0.438
Dyslipidemia		2101 (70%)	1879 (69%)	222 (77%)	0.009
Premature CAD		446 (15%)	405 (15%)	41 (14%)	0.726
Hypertension		2433 (81%)	2183 (80%)	250 (86%)	0.016
Heart failure		441 (15%)	384 (14%)	57 (20%)	0.012
Diabetes mellitus		1903 (63%)	1738 (64%)	165 (57%)	0.018
Stroke/TIA		274 (9.1%)	254 (9.0%)	29 (10%)	0.580
*Clinical (parameters) at presentation*					
HR, mean ± SD, bpm	86 ± 21		86 ± 21	85 ± 22	0.229
SBP, mean ± SD, mmHg	142 ± 28		142 ± 28	143 ± 29	0.519
DBP, mean ± SD, mmHg	80 ± 16		80 ± 16	80 ± 17	0.800
Crea, p50 (IQR), µmol/L	86 (68–113)		86 (68–113)	85 (68–104)	0.772
LVEF, mean ± SD, %	49 ± 13		48 ± 13	50 ± 13	<0.001
GRACE risk, mean ± SD	130 ± 42		130 ± 42	130 ± 43	0.789
CRUSADE risk score	38 ± 15		38 ± 15	38 ± 15	0.735
Major bleed	62 (2.1%)		54 (2.0%)	8 (2.8%)	0.380
*Killip class*					0.320
I—no heart failure	2270 (75%)		2063 (76%)	207 (71%)	
II—rales	457 (15%)		403 (15%)	54 (19%)	
III—pulmonary edema	250 (8.3%)		224 (8.2%)	26 (9.0%)	
IV—cardiogenic shock	30 (1.0%)		27 (1.0%)	3 (1.0%)	
*Discharged diagnosis* *					<0.001
LBBB MI	19 (0.7%)		16 (0.6%)	3 (1.1%)	
NSTEMI	1474 (51%)		1358 (52%)	116 (41%)	
STEMI	476 (17%)		433 (17%)	43 (15%)	
Unstable angina	908 (32%)		789 (30%)	119 (42%)	

SD, standard deviation; BMI, body mass index; MI, myocardial infarction; CAD, coronary artery disease; TIA, transient ischemic attack; HR, heart rate; bpm, beats per minute; SBP, systolic blood pressure; DBP, diastolic blood pressure; Crea, first serum creatinine; p50, median; IQR, interquartile range; LVEF, left ventricular ejection fraction; GRACE, global registry of acute coronary events; LBBB, left bundle branch block; NSTEMI, non-ST myocardial infarction; STEMI, ST-elevation myocardial infarction. BMI was missing in 47 subjects, HR in 4 subjects, SBP and DBP in 5 subjects, creatinine in 10 subjects, LVEF was missing in 468 subjects, GRACE in 14 subjects, 64 subjects in CRUSADE risk score. * The ‘discharged diagnosis’ excluded 129 patients that died in-hospital during the index admission while 1 patient had ‘discharged diagnosis’ missing. Percentages might not add up to 100% due to rounding off.

**Table 2:** Medication utilization of the acute coronary syndrome cohort stratified by non-steroidal anti-inflammatory drug (NSAID) use.

Characteristic, *n (%) Unless Specified Otherwise*	All (N = 3007)	NSAID Use		*p*-Value
		No (n = 2717)	Yes (n = 290)	
*Prior medications*				
Aspirin	2397 (80%)	2151 (79%)	246 (85%)	0.023
Clopidogrel	863 (29%)	762 (28%)	101 (35%)	0.015
ACEIs	1562 (52%)	1437 (53%)	125 (43%)	0.002
ARBs	573 (19%)	485 (18%)	88 (30%)	<0.001
Beta blockers	1828 (61%)	1639 (60%)	189 (65%)	0.108
Statins	2428 (81%)	2186 (80%)	242 (83%)	0.219
Other LLDs	60 (2.0%)	57 (2.1%)	3 (1.0%)	0.273
Oral nitrates	1049 (35%)	670 (42%)	379 (27%)	<0.001
CCBs	599 (20%)	523 (19%)	76 (26%)	0.005
H2-receptor antagonists	410 (14%)	331 (12%)	79 (27%)	<0.001
Proton pump inhibitors	617 (21%)	527 (19%)	90 (31%)	<0.001
*Discharged medications (N = 2747)* *				
Aspirin	2645 (96%)	2388 (96%)	257 (98%)	0.197
Clopidogrel	2009 (73%)	1792 (72%)	217 (83%)	<0.001
ACEIs	1795 (65%)	1624 (65%)	171 (65%)	0.907
ARBs	499 (18%)	443 (18%)	56 (21%)	0.168
Beta blockers	2324 (85%)	2089 (84%)	235 (89%)	0.025
Statins	2675 (97%)	2414 (97%)	261 (99%)	0.050
Other LLDs	75 (2.7%)	67 (2.7%)	8 (3.0%)	0.745
Oral nitrates	1722 (63%)	1562 (63%)	160 (61%)	0.509
CCBs	509 (19%)	462 (19%)	47 (18%)	0.770
Dual antiplatelets	2705 (98%)	2445 (98%)	260 (99%)	0.589
5-drug regimen	1427 (52%)	1265 (51%)	162 (62%)	0.001
H2-receptor antagonists	582 (21%)	517 (21%)	65 (25%)	0.142
Proton pump inhibitors	356 (13%)	315 (13%)	41 (16%)	0.183

ACEI, angiotensin-converting enzyme inhibitor; ARB, angiotensin II receptor blocker; LLD, lipid lowering drug; CCBs, calcium channel blockers; Dual antiplatelets, aspirin and clopidogrel concurrently; 5 drug regimen, concurrent prescribing of aspirin, clopidogrel, ACEI/ARB, statin, beta blocker. The discharged medications excluded patients that died in-hospital (n = 129) during the index admission as well as patients that had missing drug information (n = 131), while patients on discharged ARB, statins, other LLTs, oral nitrates, CCBs, proton pump inhibitors and H2 receptor blockers had one further patient excluded due to missing drug information. Percentages might not add up to 100% due to rounding off.

**Table 3:** Association between non-steroidal anti-inflammatory drug (NSAID) use and 12-month cumulative major adverse cardiovascular events (MACE) in acute coronary syndrome patients in the Arabian Gulf.

Outcome	Univariate Statistics (NSAID Use)				Multivariate Logistic Regression			
	All (N = 2910)	No (n = 2627)	Yes (n = 283)	*p*-Value	Adj. OR [95% CI]	Adj. *p*-Value	HL	ROC
Stroke/TIA	143 (4.9%)	115 (4.4%)	28 (9.9%)	<0.001	2.50 [1.51–4.14]	<0.001	0.917	0.75
Myocardial infarction	249 (8.6%)	223 (8.5%)	26 (9.2%)	0.690	1.26 [0.80–1.99]	0.320	0.102	0.71
All-cause Mortality	395 (13.6%)	366 (14.0%)	29 (10.3%)	0.086	0.79 [0.46–1.34]	0.383	0.075	0.78
Re-admissions	823 (28.3%)	705 (26.8%)	118 (41.7%)	<0.001	2.09 [1.59–2.74]	<0.001	0.665	0.61
Total MACE	1195 (41.1%)	1052 (40.1%)	143 (50.5%)	0.001	1.89 [1.44–2.48]	<0.001	0.391	0.67

Adj. OR, adjusted odds ratio; CI, confidence interval; HL, Hosmer–Lemeshow *p*-value; ROC, area under the receiver operating curve (also known as *c*-statistic); TIA, transient ischemic attack; re-admissions, re-admissions for cardiac reasons. MACE included stroke/TIA, myocardial infarction, mortality and re-admissions for cardiac reasons. For 6-month and 12-month follow-up, the events were cumulative. Multivariate analyses were conducted using logistic regression models utilizing the simultaneous method. The covariates in the models included GRACE risk score (derived from age, heart rate, systolic blood pressure, serum creatinine, cardiac arrest at admission, ST segment deviation on EKG, abnormal cardiac enzymes and Killip class) as well as gender, smoking status, marital status, employment status, education status, body mass index, diabetes mellitus, peripheral artery disease, percutaneous coronary intervention/coronary artery bypass graft, prior event and use of evidence-based cardiac medications at hospital discharge (aspirin, clopidogrel, beta blocker, statin, angiotensin converting enzyme inhibitor (ACEI) or angiotensin receptor blocker (ARB)). Over the 1-year follow-up period, there were losses to follow-up of 97 (3.2%) patients.

**Table 4:** Demographic and clinical characteristics between the non-steroidal anti-inflammatory drug group remaining at the end of the year and the cohort that was lost to follow-up (LTF).

Characteristic, *Mean ± SD Unless Specified Otherwise*	LTF (n = 97) 3.2%	Remaining (n = 2910) 96.8%	*p*-Value
*Demographic*			
Age, years	62 ± 11	62 ± 12	0.940
Female gender, n (%)	38 (39%)	1118 (38%)	0.880
BMI, kg/m^2^	29.4 ± 5.5	29.1 ± 7.1	0.646
*Clinical, n (%)*			
Prior MI	32 (33%)	994 (34%)	0.811
Hypertension	81 (84%)	2352 (81%)	0.509
Diabetes mellitus	69 (71%)	1834 (63%)	0.103
Stroke/TIA	8 (8.3%)	266 (9.1%)	0.764
*Presentation, n (%)*			
SBP, mmHg	143 ± 29	142 ± 28	0.960
DBP, mmHg	80 ± 17	80 ± 16	0.832
Killip ≥ 2, n (%)	23 (24%)	714 (25%)	0.853
GRACE risk score	132 ± 41	130 ± 42	0.668
CRUSADE risk score	39 ± 15	38 ± 15	0.603
Major bleeding	3 (3.1%)	59 (2.0%)	0.451
Prior PCI	30 (31%)	793 (27%)	0.424

SD, standard deviation; BMI, body mass index; MI, myocardial infarction; TIA, transient ischemic attack; SBP, systolic blood pressure; DBP, diastolic blood pressure; GRACE, global registry of acute coronary events; PCI, percutaneous coronary intervention (includes any prior PCI). BMI was missing in 47 subjects, SBP and DBP in 5 subjects, GRACE risk score in 14 subjects and 64 subjects in the CRUSADE risk score. Percentages may not add up too 100% due to rounding off.

## Data Availability

Data were anonymized for this study. All data are available upon request to the corresponding author.
